# The importance of comorbidity in analysing patient costs in Swedish primary care

**DOI:** 10.1186/1471-2458-6-36

**Published:** 2006-02-16

**Authors:** Sven G Engström, Lennart Carlsson, Carl-Johan Östgren, Gunnar H Nilsson, Lars A Borgquist

**Affiliations:** 1 Ryd primary health care centre, Linköping, Sweden; 2 General Practice, Department of Health and Society, Faculty of Health Sciences, Linköping University, Sweden; 3 The Neurotec Department, Center for Family and Community Medicine, Karolinska Institutet, Stockholm, Sweden; 4 Ödeshög primary health care centre, Ödeshög, Sweden

## Abstract

**Background:**

The objective was to explore the usefulness of the morbidity risk adjustment system Adjusted Clinical Groups^® ^(ACG), in comparison with age and gender, in explaining and estimating patient costs on an individual level in Swedish primary health care. Data were retrieved from two primary health care centres in southeastern Sweden.

**Methods:**

A cross-sectional observational study. Data from electronic patient registers from the two centres were retrieved for 2001 and 2002, and patients were grouped into ACGs, expressing the individual combination of diagnoses and thus the comorbidity. Costs per patient were calculated for both years in both centres. Cost data from one centre were used to create ACG weights. These weights were then applied to patients at the other centre. Correlations between individual patient costs, age, gender and ACG weights were studied. Multiple linear regression analyses were performed in order to explain and estimate patient costs.

**Results:**

The variation in individual patient costs was substantial within age groups as well as within ACG weight groups. About 37.7% of the individual patient costs could be explained by ACG weights, and age and gender added about 0.8%. The individual patient costs in 2001 estimated 22.0% of patient costs in 2002, whereas ACG weights estimated 14.3%.

**Conclusion:**

ACGs was an important factor in explaining and estimating individual patient costs in primary health care. Costs were explained to only a minor extent by age and gender. However, the usefulness of the ACG system appears to be sensitive to the accuracy of classification and coding of diagnoses by physicians.

## Background

Accurate methods are needed for estimating the burden of morbidity in a population to enable resource allocation corresponding to the health care needs of the population. This is particularly important in relation to the funding of primary health care (PHC) centres, as there is greater variation in terms of need between localities than between regions or counties [1]. Determinants of health care utilisation and health care costs are of great interest in this context, and a number of factors have been identified in empirical investigations [2]. In a study from Canada, age and gender explained only 5–9% of the variation in health care expenditures [3]. The influence of morbidity and of socio-economic factors has also been investigated. The studies showed that there was substantial variation in hospital admission rates among general practitioners (GPs) due to socio-demographic patient factors associated with deprivation [4,5].

In countries with a national health care system, where public authorities are responsible for health care provision in regions and smaller areas, interest has focused on demographic and socio-economic determinants of health care needs. In this regard, the underprivileged area score (UPA), or the Jarman index, was developed in the UK [6]. The index has been adapted to Swedish conditions, slightly modified, and renamed the Care Need Index (CNI) [7].

In countries with a health care system primarily based on individual health insurance, interest has focused on measures based on individual patients' characteristics. In the USA, several instruments have been developed to compensate for differences in case-mix and to adjust for differences in risk [3]. One of these instruments is the Adjusted Clinical Groups® (ACG) system, which assigns patients to morbidity categories based on disease patterns and expected resource requirements [8,9]. The grouping algorithm enables each diagnosis to be classified as one out of 32 types of morbidity (Aggregated Diagnosis Groups), depending on five combined criteria: i) likely persistence of the condition, ii) grade of severity, iii) aetiology, iv) diagnostic certainty, and v) need for speciality care. Thus each ACG is used as an estimate for a group of patients with the same constellation of morbidities, thereby indicating the need for care of each category of patients. Research, development and documentation of the ACG system has been performed mainly in the USA [8-12] and Canada [3,13-15], and only a few European studies of the ACG system have been published [16-19].

The ability of the ACG system to measure individual comorbidity and the burden of morbidity in a population is of special interest from a PHC perspective, since a GP cares for a number of individual patients who often suffer from several diseases, all of which are normally treated by the GP. A retrospective study was conducted on encounter data from a three-year period in a Swedish county council and it is published in this issue of BMC Public Health.

The aim of this study is to explore the usefulness of the ACG system, in comparison with age and gender, in explaining and estimating patient costs on an individual level in a Swedish PHC setting.

## Methods

### Setting

Two PHC centres in the county of Östergötland in southeastern Sweden were included in the study. Ödeshög PHC centre is situated in a rural municipality with about 5600 inhabitants. Ryd PHC centre has a registered patient population of about 9000 and is located in the city of Linköping, with about 130 000 inhabitants. The two PHC centres will be referred to as Ödeshög and Ryd. Both are complete Swedish PHC centres with GPs, district nurses, physiotherapists, occupational therapists and social workers. Both use electronic patient record (EPR) systems – BMS^® ^in Ödeshög, and Swedestar^® ^in Ryd. The Swedish PHC version of the International Classification of Diseases and Related Health Problems (ICD-10) was used for labelling health problems [20]. Ödeshög was involved in this study to create ACG weights, and Ryd was used to explore the usefulness of the ACG system.

### Data retrieval

Data from the EPRs of all patients at both PHC centres for 2001 and 2002 that were used in this study included: an encrypted identity number, age, gender, numbers and types of encounters, category of caregiver and diagnoses.

### Cost calculations

The *Patient-level Clinical Costing *method yields information about health care costs on an individual level, and was initially used within hospital care in Sweden. The first application of this costing method in PHC was done at Ödeshög [21][22]. Data on costs per patient in 2001 and 2002 from that study were used for the patients at Ödeshög. In order to estimate individual patient costs at Ryd, data from all patient contacts (i.e. both direct and indirect contacts) during 2001 and 2002 were extracted from the EPRs at Ryd, including an encrypted identification number for each patient, the profession of the caregiver, contact date and type of contact (e.g. face-to-face encounter, telephone, house call or contact through a third party), and these contacts were priced according to their resource consumption. This included costs for the time consumed of all kinds of personnel, localities, computer systems, and overhead costs, together with all kinds of laboratory service, and x-ray, but not including costs for other referrals or drugs.

The yearly cost per patient was subsequently calculated by adding all the costs for all contacts in PHC for that patient during each year.

All costs were calculated in Swedish crowns (SEK) (exchange rate June 2001: 1 Euro = SEK 9.20).

### ACG grouping

Only patients with at least one registered IDC-10 code during a calendar year were included in the ACG grouping, and consequently only these patients were included in the creation and application of ACG weights. Data on patients at both PHC centres with registered diagnoses during 2001 and 2002 were extracted from the EPRs. The patients were assigned to ACGs using version 6.0 of the ACG software [23]. This is constructed to use the codes from the International Classification of Diseases, Ninth Revision (ICD-9), and the classification in use (ICD-10) was therefore mapped to ICD-9 codes by use of cross-mapping tables from the WHO [24].

### Creating ACG weights

The data on individual patient costs from Ödeshög were used to create the ACG weights. Patients with costs more than three standard deviations above the mean were excluded in order to prevent a few extremely costly patients from producing a false high mean cost for some ACGs. The calculation of weights was done by using each individual patient's costs for 2001 and for 2002 at Ödeshög, which were then applied to the categories of patients in terms of ACGs. Cost data from both years were used in order to get a sufficient number of individual yearly costs in each ACG. Relative ACG weights were then calculated and defined as the mean cost for each ACG divided by the mean cost for all ACGs.

### Application of ACG weights

The relative weights of ACGs (from Ödeshög) were assigned to each patient at Ryd based on that patient's individual ACG assignment. Patients at Ryd with costs more than three standard deviations above the mean were excluded. The usefulness of ACG in explaining individual patient costs was explored in a concurrent model by comparing data from Ryd separately for 2001 and 2002.

The relative ACG weights applied (from Ödeshög, based on both 2001 and 2002) were compared with ACG weights from Ryd, from the USA (a database at Johns Hopkins University) [23], and from Canada (two sites, Manitoba and British Columbia) [3] in the concurrent model.

The usefulness of ACG in estimating patient costs for individuals was explored in a prospective model by comparing data from 2001 with costs from 2002 at Ryd.

### Statistics

Spearman's correlation coefficient (Spearman's rho) was used for bivariate correlation for each year between the variables age, gender and ACG weights in a concurrent model. Stepwise multiple linear regression analysis was then used, for each year, to explore the variation of individual patient costs with age, gender, and ACG weights as the independent variables.

In order to estimate the predictive usefulness on an individual level in a prospective model, another stepwise multiple linear regression analysis was performed with the individual patient costs in 2002 as the dependent variable and age, gender, ACG weights in 2001, and individual patient costs in 2001 as the independent variables.

The statistical software SPSS^® ^version 11.5 was used.

## Results

The mean cost per patient at Ödeshög was higher compared with Ryd both in 2001 and 2002 (Table 1). At both PHC centres the variation in individual patient costs within age groups was considerable in both years. The mean cost for women was about 30% higher than for men. The number of patients, both those with and those without a registered diagnosis, increased substantially in 2002.

Data from Ödeshög were used for the creation of ACG weights. Thus there were 3 073 patients diagnosed by a GP in 2001, and 3 144 patients in 2002, who were grouped by the ACG system (Table 1). These relative ACG weights ranged from 0.3001 to 4.1798. Data from Ryd were used for the evaluation of ACG weights. There were 4 478 patients diagnosed by a GP in 2001 and grouped by the ACG system. A total of 5 163 patients had an encounter with the PHC centre at Ryd during this year. The variation in individual patient costs within each ACG was substantial (Table 2). As can be seen in the table, the very high relative weights for ACG # 4420, a group of patients with a constellation of 4–5 types of morbidities, correspond to the very highest mean costs per patient.

The individual patient costs and other variables at Ryd showed a correlation of 0.633 (Spearman's rho) for ACG weights, 0.308 for age, and 0.119 for gender in 2001 in the concurrent model. In 2002 the results were similar. Age, gender and ACG weights (as independent variables in a stepwise multiple regression analysis) together explained 38.5% of the individual patient costs in 2001 (Table 3), and this figure was 34.3% in 2002. In 2001, ACG weights explained 37.7% of the variance in the concurrent model, while age and gender added 0.8%. Age and gender alone explained 11.4%. In 2002 the results were similar.

Replacing the ACG weights from Ödeshög with relative weights from other areas resulted in about the same or lower adjusted R-square values. These weights yielded the following values: 0.377 (Ryd), 0.203 (USA), 0.320 (Manitoba, Canada), and 0.337 (British Columbia, Canada).

The costs of individual patients in 2001 turned out to be the most important factor, by 22.0%, for estimating the individual patient costs at Ryd in 2002 in the prospective model (Table 4). The ability of the ACG weights alone to estimate patient costs next year was 14.3%. The gender factor did not affect the result to any degree and thus has been omitted in the table.

## Discussion

In this study the ACG weights were found to be a major factor in a concurrent model, explaining patient costs during one and the same year. Age and gender explained about 11% of the individual patient costs, and when the ACG weights were added, the explanatory ability was improved and reached 38.5%. The ACGs with more complex constellations of types of morbidity were generally more resource consuming, indicating the influence of comorbidity. When estimating costs the next year in a prospective model, the ACG weights constituted the second most important factor, while the costs the preceding year were the major factor.

It can be argued that costs from year one should not be used as a factor to estimate costs in year two in a prospective model, since most costs at a PHC centre are relatively stable. In this case, though, we studied not only costs on ACG group level, but also the yearly costs of individual patients. Thus it is worthwhile to include prior costs as a variable in the analysis in order to reflect the year to year variability in the individual patient's costs, when evaluating the potential of the ACGs in the prospective model.

The results in this study were in accordance with findings in a Canadian study based on physician claims, where age and gender explained 9% of the variation in costs, a figure that increased to 53% when Aggregated Diagnosis Groups were added [3]. In a Spanish study in which prospective registration of diagnoses that was independent of registration of other medical information, and in which the number of GP consultations (without cost estimation) was used as outcome measure, the corresponding figures were 7% for age and gender and 50% when ACGs were included [18]. The ability of the ACGs to estimate about 15% of individual patient costs in the following year in our study was in line with a claims-based study in the USA [15].

With respect to the cost calculations, the contact-based analysis of individual patient costs can be considered sufficiently detailed to provide a reliable distribution of costs between patients. Registration of patient contacts in the EPR was a prerequisite for enabling the caregiver to record medical information, and consequently the drop-out rate was low.

The original diagnoses were coded in ICD-10 and converted to ICD-9-codes. The crossover mapping was checked manually to ensure that it was as precise as possible. The fact that ACG grouping is not based on the number of encounters with a specific diagnosis, but on the mere appearance of a diagnosis (once or on several occasions during a whole year), makes the ACG system less sensitive to variations in the completeness of diagnosis recording. The effects of physician preference are further reduced, since the ACG system categorises diagnoses in only 32 types of morbidity (the ADGs).

In our study the ACG weighting appears to be sensitive to the accuracy with which physicians enter diagnoses into the EPRs. The coding situation in PHC in Sweden is reported to be quite accurate, but there is still a need for improved quality [25]. Due to the organisation of the EPRs at Ryd, a more complete registration of diagnoses was done there than at Ödeshög, resulting in a higher mean ACG weight albeit lower mean patient costs.

To explain the influencing factors in the concurrent model in more detail, it could be appropriate to analyse the case-mix of patients at Ryd a bit further. As can be seen in Table 1, the mean ACG weight increased significantly from year one to year two, as did the number of patients grouped, although the mean costs per patient were lowered. Also, the proportion of females was higher the second year along with a decline in mean age. However, these factors did not affect our results in the prospective model, as we used the subset of patients being diagnosed both years as the basis for the estimate.

The creation of ACG weights was limited by the fact that not all patients (i.e. only those with at least one diagnosis per year) were included. However, since the costs of all patients without any diagnosis constituted no more than 2.5% of the total, this limitation probably affects our results to only a minor degree. A further limitation of the ACG weights was that the population of Ödeshög was small. This was mitigated to some extent by aggregating data from two consecutive years. Nevertheless, there were still some ACGs with only a few patients, making these weights uncertain. Constructing weights for a larger population might have resulted in greater explanatory ability. However, weights provided by the ACG software [23], based on a reference population of 2 000000 subjects in the USA, gave an adjusted R-square as low as 0.20 in our population, compared to Canadian weights from Manitoba and British Columbia with an adjusted R-square of 0.32 and 0.34, respectively. Hence, data from the Swedish PHC centre in Ödeshög were found to yield more useful relative weights for a Swedish PHC setting compared to weights based on statistically well-founded data from the USA and Canada.

When exploring the predictive usefulness, the individual-based approach is limited by the fact that it is based on a sub-population of individuals (i.e. only patients who are included both years), which is not fully representative for the whole population. However, our figures give an idea of the usefulness of the ACG weights in estimating individual patient costs, as well as an idea of the continuity of individual patient costs.

Efforts to construct a more comprehensive database on costs per patient in PHC are ongoing, based on the same population as reported in the adjacent article (with PHC data for three years from a Swedish county) in this issue of BMC Public Health. That analysis might lead to a higher degree of certainty when using relative ACG weights. The possibility of integrating data on drug prescriptions, as is indicated in a new version of the ACG grouping software, might improve the usefulness of the system in concurrent as well as prospective models.

It would have been advantageous to test the significance of a socio-economic variable, such as the Care Need Index, in explaining and estimating patient costs [26]. However, the CNI was not applicable in our study because of the small number of individuals at Ryd, and because of the skewed distribution of CNI scores, since 85% of the patients were found in only five indexed areas. Individual socio-economic data were not available in our study, but will probably be needed in order to explain and estimate individual patient costs more accurately. Further, there is need for more research and development in this area, also integrating other variables such as functional and self-perceived health status measures.

In the future, measures will be required to reduce the variation and enhance the quality of diagnostic coding in PHC. However, it is unrealistic to expect that this problem can be totally eliminated, as the criteria used for diagnostic labelling by different physicians have been shown to vary [27]. Thus, introduction of methods that might compensate for variations in the completeness of physicians' registration of diagnoses could increase the usefulness of the ACG system.

## Conclusion

The comorbidity, expressed by the individual constellation of morbidities in the ACG system, was associated with a large proportion of the variation in PHC costs. Age and gender could explain individual patient costs to a minor degree. Accordingly, the ACG system has the potential to be a useful instrument for describing and explaining concurrent resource consumption in PHC, even on the PHC centre level, as well as for estimating future costs for health care in a prospective model. As the ACG system appears to be sensitive regarding the accuracy with which physicians register diagnoses, measures will be required to reduce this variation and enhance the quality of diagnostic coding in PHC. Individual socio-economic data, as well as other health related measures, will probably be needed in future studies in order to reinforce the explanation and estimation of individual patient costs.

## Competing interests

The authors declare that they have no competing interests.

## Authors' contributions

SGE collected the data and carried out the first calculations of the results, and drafted the manuscript. LC handled the ACG software, rechecked and revised the calculations and drafted the manuscript. CJÖ collected the data from Ödeshög, participated in the statistical analysis and drafted the manuscript. GHN sketched the design of the study, drafted the manuscript and helped to co-ordinate the work. LAB participated in the design of the study, collected the data on ACG weights, drafted the manuscript and co-ordinated the work.

All authors read and approved the final manuscript.

## Pre-publication history

The pre-publication history for this paper can be accessed here:



## Figures and Tables

**Table 1:** Characteristics of patients at Ryd and Ödeshög PHC centres

	Ödeshög		Ryd	
	2001	2002	2001	2002
		
Number of patients				
- enrolled	5 600	5 600	9 000	9 000
- contacting the PHC	4 075	4 122	5 163	6 539
- diagnosed by a GP	3 073	3 144	4 478	5 358
Number of diagnoses/patient	2.07	2.04	2.28	2.17
Mean age	45.8	46.9	36.4	35.9
Proportion of females	54.2	53.3	52.1	57.9
Mean costs per patient (SEK)*	3 331	3 293	2 356	2 265
Mean ACG weight	1.0 **		1.07	1.19

* Swedish Crowns

** 1.0 since the individual patients costs for the two years together were the base for the weights

**Table 2:** The most frequent ACGs, distribution of patients, mean and range for costs per patient in Swedish crowns (SEK) at Ryd PHC centre in 2001. (N = 4478)

ACG	ACG	Costs per patient (SEK)			
		
Group	Description	N	Mean	Range	
0300	Acute minor, age 6+	646	1 048	90 – 15 755	
4100	2–3 other ADG combinations, age >34	555	3 812	153 – 44 693	
0500	Likely to recur without allergy	458	1 048	90 – 12 905	
0400	Acute major	356	1 480	50 – 14 773	
0900	Chronic medical, stable	307	1 542	90 – 19 271	
2100	Acute minor & Likely to recur	174	2 120	90 – 13 455	
1800	Acute minor & Acute major	160	2 540	530 – 27 116	
2300	Acute minor & Chronic medical, stable	115	2 416	106 – 11 332	
2800	Acut major & Likely to recur	103	2 613	220 – 34 406	
0800	Chronic medical, unstable	102	2 630	90 – 16 034	
1600	Preventive / Administrative	98	978	90 – 4 267	
4420	4–5 ADG, 1 major, age >44	93	6 523	683 – 33 465	
	Total	3 167			

ACG	Relative weights				
	
Group	Ödeshög	Ryd	USA	Manitoba	British Columbia

0300	0.55	0.39	0.18	0.23	0.27
4100	1.33	1.47	1.11	0.97	0.79
0500	0.46	0.38	0.25	0.26	0.21
0400	0.61	0.56	0.42	0.35	0.27
0900	0.60	0.58	0.33	0.37	0.34
2100	0.95	0.81	0.43	0.51	0.56
1800	1.06	0.95	0.71	0.60	0.72
2300	1.02	0.95	0.53	0.60	0.63
2800	1.11	1.00	0.91	0.72	0.54
0800	0.96	0.98	1.02	0.59	0.49
1600	0.48	0.34	0.14	0.22	0.14
4420	2.06	2.56	2.76	1.85	1.53

**Table 3:** Multiple linear stepwise regression analysis with individual costs per patient in 2001 at Ryd PHC centre as dependent variable, concurrent model

**Adjusted R^2 ^= 0.385**		95% CI of b-coefficient		
			
Independent variables	Unstandardised b-coefficient	Lower	Upper	P-value
ACG weights	1 974	1 886	2 062	<0.001
Age	8	6	11	<0.001
Gender	-215	-315	-116	<0.001

**Table 4:** Multiple linear stepwise regression analysis with individual costs per patient in 2002 at Ryd PHC centre as dependent variable, prospective model

**Adjusted R^2 ^= 0.267**		95% CI of b-coefficient		
			
Independent variables 2001	Unstandardised b-coefficient	Lower	Upper	P-value
Costs	0.28	0.25	0.31	<0.001
ACG weights	599	455	743	<0.001
Age	16	13	20	<0.001
